# Nuclear genome organization in fungi: from gene folding to Rabl chromosomes

**DOI:** 10.1093/femsre/fuad021

**Published:** 2023-05-17

**Authors:** David E Torres, Andrew T Reckard, Andrew D Klocko, Michael F Seidl

**Affiliations:** Theoretical Biology and Bioinformatics, Department of Biology, Utrecht University, Padualaan 8, 3584 CH Utrecht, The Netherlands; Laboratory of Phytopathology, Wageningen University and Research,Droevendaalsesteeg 4, 6708 PB Wageningen, The Netherlands; Department of Chemistry and Biochemistry, University of Colorado Colorado Springs, 234 Centennial Hall, 1420 Austin Bluffs Pkwy, Colorado Springs, CO 80918 USA; Department of Chemistry and Biochemistry, University of Colorado Colorado Springs, 234 Centennial Hall, 1420 Austin Bluffs Pkwy, Colorado Springs, CO 80918 USA; Theoretical Biology and Bioinformatics, Department of Biology, Utrecht University, Padualaan 8, 3584 CH Utrecht, The Netherlands

**Keywords:** fungi, 3D genome organization, nuclear organization, topologically associated domain, chromosome conformation capture followed by high-throughput sequencing (Hi-C)

## Abstract

Comparative genomics has recently provided unprecedented insights into the biology and evolution of the fungal lineage. In the postgenomics era, a major research interest focuses now on detailing the functions of fungal genomes, i.e. how genomic information manifests into complex phenotypes. Emerging evidence across diverse eukaryotes has revealed that the organization of DNA within the nucleus is critically important. Here, we discuss the current knowledge on the fungal genome organization, from the association of chromosomes within the nucleus to topological structures at individual genes and the genetic factors required for this hierarchical organization. Chromosome conformation capture followed by high-throughput sequencing (Hi-C) has elucidated how fungal genomes are globally organized in Rabl configuration, in which centromere or telomere bundles are associated with opposite faces of the nuclear envelope. Further, fungal genomes are regionally organized into topologically associated domain-like (TAD-like) chromatin structures. We discuss how chromatin organization impacts the proper function of DNA-templated processes across the fungal genome. Nevertheless, this view is limited to a few fungal taxa given the paucity of fungal Hi-C experiments. We advocate for exploring genome organization across diverse fungal lineages to ensure the future understanding of the impact of nuclear organization on fungal genome function.

## Teaser

Within the nucleus, fungal genomes are organized hierarchically, from the association of chromosomes to topological structures at individual genes, and this organization directly impacts genome function.

## Introduction

In eukaryotes, the DNA within the nucleus is organized as chromatin, a dynamic complex formed by DNA in association with proteins (Campos and Reinberg 2009). Approximately 146 bp of DNA is wrapped around an octamer of histone proteins, two copies of histones H2A, H2B, H3, and H4, that together form the nucleosome core particle (Luger et al. 1997), which represents the smallest organizational unit of chromatin (Campos and Reinberg 2009). DNA and histone proteins are subjected to a plethora of chemical modifications such as, but not limited to, methylation or acetylation of lysine residues in histone proteins, and specific combinations of these modifications—the histone code—have been associated with the formation and maintenance of, as well as dynamic transitions between, different local chromatin organization (Strahl and Allis 2000, Jenuwein and Allis 2001). Depending on its local compaction state, chromatin can be broadly subdivided into two types where the DNA is more (euchromatin) or less (heterochromatin) accessible to the transcriptional machinery, thereby directly influencing gene expression (Bannister and Kouzarides 2011, Allshire and Madhani 2018). Euchromatin is considered to be transcriptionally active, while heterochromatin is typically transcriptionally silent (Jenuwein and Allis 2001). Heterochromatin can be further subdivided into constitutive heterochromatin, i.e. largely devoid of genes, and facultative heterochromatin that typically contains genes that are transcriptionally repressed during specific developmental or environmental conditions (Trojer and Reinberg 2007, Saksouk et al. 2015, Wang et al. 2016, Allshire and Madhani 2018).

Local chromatin can be folded in the three-dimensional (3D) space of the nucleus to structurally organize and package DNA, while also enabling precise gene expression (Lieberman-Aiden et al. 2009, Sexton and Cavalli 2015, Bonev and Cavalli 2016, Hoencamp et al. 2021). For instance, higher-order chromatin structures allow the spatial association of genomic elements that are physically separated on the linear DNA strand or occur on different chromosomes, and conversely such structures can physically segregate nearby genomic sites through specific folding barriers (Tolhuis et al. 2002, West and Fraser 2005, Lieberman-Aiden et al. 2009). Consequently, the disruption of DNA folding can result in ectopic interactions of genes and their regulatory regions (Sexton and Cavalli 2015, Bonev and Cavalli 2016, Krumm and Duan 2019), for instance, by hijacking enhancers as observed for proto-oncogenes (Northcott et al. 2014, Flavahan et al. 2016). Consequently, it is important to understand how the 3D genome organization in eukaryotes is established, how it affects genome function, and how this chromatin organization and the processes that establish it are conserved between eukaryotes. While much about genome organization and its function is known in a few animal and plant model systems, there is a dearth of knowledge about these processes in most eukaryotes, including fungi.

The fungal kingdom is estimated to contain millions of different species (Blackwell 2011). Fungi are important components of worldwide ecosystems as decomposers of organic material derived from plants and animals, and many have been exploited for decades in food production and biotechnology (Stajich et al. 2009). Many fungi are also symbionts that can engage in mutualistic to parasitic interactions with other organisms, which can have significant economic and ecological impact (Fisher et al. 2012, 2020). For instance, in agriculture, plant pathogenic fungi can cause devastating epidemics in staple and commodity crops necessary for the survival of billions of humans (Fisher et al. 2020). Importantly, many fungi are also outstanding model systems for higher eukaryotes, including humans; essential eukaryotic functions are often conserved in fungi, yet fungi are typically more simplistic, genetically tractable, and research is cost-efficient. In the last decade, research into chromatin and the organization of fungal genomes has largely focused on the bakers’ yeast *Saccharomyces cerevisiae* and fission yeast *Schizosaccharomyces pombe*, given their ease of study and the plethora of available mutant strains and genetic tools. Due to the ecological and economic relevance of fungi, it is important to further advance research into the function of fungal genomes with the goal of elucidating the regulation of gene expression and discovering how this regulatory control is influenced by nuclear genome organization. Here, we review the current knowledge of fungal genome organization, organized hierarchically from topological structures at the level of individual genes through the association of chromosomes in the nucleus to form the Rabl chromosome conformation, in which centromeres and telomeres cluster distinctly at the nuclear periphery (Box 1). Recent work in fungal model systems and increasingly in more diverse fungi has started to elucidate pertinent subnuclear chromatin structures that will be pivotal to our understanding of the function and conservation of genome organization and will provide the framework to detail how nuclear processes impact genome functions in fungi. To date, only a few high-resolution fungal Hi-C datasets are available, but an increasing number of datasets with lower resolution (>5 kb) from a wider diversity of fungi now enable researchers to draw general conclusions about hierarchical 3D structures observed that organize the fungal genome.

## The composition of fungal genomes

Over the last few decades, advances in genome sequencing technologies have provided a wealth of novel insights about the genome composition of fungi, which is a prerequisite to study nuclear genome organization in detail. Yeasts are arguably the most well-studied fungal model organisms (Botstein and Fink 2011, Liti 2015), in part due to their relatively small and well-characterized genomes. For example, the genome of the budding yeast *S. cerevisiae* is ∼12 Megabases (Mb) in size divided into 16 chromosomes, while the fission yeast *S. pombe* has a 13 Mb genome divided over only three chromosomes (Goffeau et al. 1996, Wood et al. 2002, Engel et al. 2014). Relative to the more simplistic yeasts, the genomes of filamentous fungi are typically larger and more complex, but vastly smaller than those of higher metazoans whose genomes often comprise billions of base pairs. Specifically, the genomes of filamentous fungi are typically 30–50 Mb in size, divided into variable chromosome numbers (Mohanta and Bae 2015), with several species’ genomes being much larger (Kiran et al. 2016, Porto et al. 2019). Several examples with nearly complete genome assemblies of more well-studied fungal organisms include the saprophyte and model organism *Neurospora crassa* [41 Mb divided into seven Linkage Groups (LG) or chromosomes], the soil-borne plant pathogen *Verticillium dahliae* (36 Mb divided into eight chromosomes), the human pathogens *Aspergillus fumigatus* (28 Mb across eight chromosomes), and *Cryptococcus neoformans* (19 Mb across five chromosomes), the wheat head blight fungus *Fusarium graminearum* (36 Mb across four chromosomes), and the grass endophyte *Epichloë festucae* (35 Mb across seven chromosomes) (Galagan et al. 2003, Loftus et al. 2005, Nierman et al. 2005, Faino et al. 2015, King et al. 2015, Winter et al. 2018, Bowyer et al. 2022).

**Figure 1.**
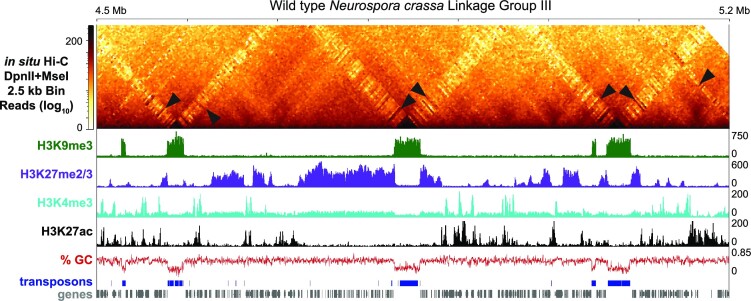
Chromatin profile and evidence for cross-compartment (heterochromatic–euchromatic) contacts in *N. crassa*. Histone post-translational modifications [both activating (H3K4me3 and H3K27ac) and repressive (H3K9me3 and H3K27me2/3)] demarcate the chromatin of *N. crassa*. Regions of the *N. crassa* genome that contain numerous transposon relicts are often rich in adenine/thymine base pairs (AT-rich) and devoid of genes. The occurrence of Hi-C interactions between euchromatin (or facultative heterochromatin) and H3K9me3-marked constitutive heterochromatin may be important for gene regulation. (Top) Corrected *in situ* Hi-C heatmap at 2.5 kb resolution showing interactions across a ∼700 kb section of the right arm on Linkage Group III. Arrowheads highlight interactions between H3K9me3-marked heterochromatic regions and euchromatin. (Bottom) The different tracks show enrichment of histone post-translational modifications based on Chromatin Immunoprecipitation sequencing (ChIP-seq) experiments (Klocko et al. 2019, 2020, Zhu et al. 2019, Bicocca et al. 2018) the calculated % guanine/cytosine bases in the genome, predicted transposable elements (Nguyen et al. 2022), and genes. Methods for Hi-C and ChIP-seq data processing and image generation are detailed in Supplemental File 1.

Fungal genomes are well-known to be highly dynamic with discrete regions enriched with polymorphisms and chromosomal rearrangements (Raffaele and Kamoun 2012, Dong et al. 2015, Möller and Stukenbrock 2017, Torres et al. 2020). These variable regions are often embedded in different chromosomes or even comprise complete chromosomes. For instance, regions in proximity to telomeres can be highly variable in budding yeast, *Aspergillus, Neurospora*, or *Magnaporthe* species where these regions display frequent presence/absence of variation as well as chromosomal rearrangements (Farman and Kim 2005, Farman 2007, McDonagh et al. 2008, Brown et al. 2010, Chang and Ehrlich 2010, Starnes et al. 2012, Jamieson et al. 2013, 2018, Yue et al. 2017). Other species like *V. dahliae* contain similar adaptive genomic regions (AGRs) that are embedded within different chromosomes and contain *in planta* expressed genes (de Jonge et al. 2012, 2013, Faino et al. 2016, Cook et al. 2020). Entire chromosomes are variable within strains of *Fusarium oxysporum* or *Zymoseptoria tritici*, as they contain genes important for the biology of these fungi (van Dam et al. 2017, Möller et al. 2019). It is becoming increasingly evident that dynamic regions in fungal genomes are often composed of highly repetitive relicts of transposable elements (Möller and Stukenbrock 2017, Frantzeskakis et al. 2019). Consequently, these regions are known to have a high number of adenine and thymine (AT) base pairs (bp), which lower the overall guanine and cytosine (GC; can include G:C base pairs or GpC/CpG dinucleotides in a single strand) content of fungal genomes from an approximately 50% GC bp composition. These AT-rich regions, termed AT-isochores to specifically delineate small genomic regions depleted of GC bp (Testa et al. 2016), can be interspersed in the core chromosomes or localized on accessory chromosomes. For example, one-third of the genome of the fungus *Leptosphaeria maculans*, a Dothideomycete pathogen of the canola plant crops for producing rapeseed oil, contains blocks of dense AT-rich sequences derived from transposable elements that often house virulence genes (Fudal et al. 2007, Rouxel et al. 2011). Similarly, dense AT- and repeat-rich regions are distributed throughout the *E. festucae* genome, comprising roughly 25% of its genome (Testa et al. 2016, Winter et al. 2018), and ∼16% of the *N. crassa* genome is interspersed with AT-rich, yet gene poor isochores (Fig. 1) (Selker et al. 2003, Lewis et al. 2009, Testa et al. 2016). Many AT-rich sequences in fungal genomes are derived from the action of repeat-induced point-mutation (RIP) (Selker and Stevens 1987, Selker 1990, Freitag et al. 2002), where the GC nucleotides in duplicated or repeated sequences are heavily mutated to contain numerous AT transition mutations, thereby inactivating the underlying sequence and increasing the local AT-content. RIP has been shown to be active in a plethora of fungal species (Cambareri et al. 1991, Clutterbuck 2011, Hane et al. 2015, Gladyshev 2017), and specifically in *Neurospora*, RIP’d transposable element relicts comprise essentially most of the AT-rich sequences across the genome (Lewis et al. 2009) (Fig. 1).

## Conserved heterochromatic region features in fungal genomes

AT-rich repetitive isochores direct the formation of the silent constitutive heterochromatin (Miao et al. 2000, Lewis et al. 2009). As initially elucidated in *Neurospora*, AT-rich isochores, by an as of yet unknown mechanism, recruit the histone methyltransferase complex DCDC (*D*IM-5/-7/-9 *C*UL4 *D*DB1^dim-8^*C*omplex) (Miao et al. 2000, Lewis et al. 2009, 2010a, Rountree and Selker 2010, Courtney et al. 2020). DCDC catalyzes the tri-methylation of lysine 9 on histone 3 (H3K9me3) of nucleosomes within AT isochores (Fig. 1), with KMT1^DIM-5^ (Lysine [*K*] *M*ethyl *T*ransferase-*1*/*D*efective *I*n *M*ethylation-*5*) being the SET domain-containing subunit with histone methyltransferase catalytic activity (Lewis et al. 2010a, b, Freitag 2017). H3K9me3 is bound by HP1 (*H*eterochromatin *P*rotein-*1*) to directly recruit the DNA methyltransferase DIM-2 for methylation on the fifth intracyclical atom of cytosine bases (5^m^C) (Nielsen et al. 2002, Freitag et al. 2004, Honda and Selker 2008). Consequently, AT-rich isochores are readily observable across fungal chromosome sequences as they appear as peaks of enrichment of H3K9me3 (Fig. 1) and cytosine methylation interspersed among the gene-rich euchromatin (Lewis et al. 2009). One important subclass of AT-rich isochores in fungal genomes are centromeres, which are critical for chromosomal function as they facilitate homologous chromosome pairing during mitosis/meiosis (Stewart and Dawson 2004, Wells et al. 2006, Krassovsky et al. 2012, Kurdzo et al. 2017, Yadav et al. 2018a, b, Previato de Almeida et al. 2019). Chromosomes of the budding yeast *S. cerevisiae* contain a “point” centromere that comprises ∼200 bp in a single nucleosome, while most other fungi have “regional” centromeres spanning thousands of base pairs (Malik and Henikoff 2009, Smith et al. 2012, Lefrançois et al. 2013, Galazka et al. 2016, Yadav et al. 2018a, b, Seidl et al. 2020). Similar to AT-rich isochores, centromeres are densely enriched for H3K9me3 and 5^m^C (Lewis et al. 2009, Smith et al. 2012, Galazka et al. 2016, Cook et al. 2020, Seidl et al. 2020), yet are differentiated from other AT-rich transposon relicts by the deposition of the centromere-specific histone variant CenH3 (Meluh et al. 1998, Malik and Henikoff 2009, Smith et al. 2012, Galazka et al. 2016, Yadav et al. 2018a, b, Seidl et al. 2020).

Facultative heterochromatin can dynamically transition between being densely compacted, thereby silencing the expression of the underlying DNA, to looser compaction resulting in chromatin being more open for activating transcription (Trojer and Reinberg 2007). Facultative heterochromatin in fungi is typically delineated by either the di- or tri-methylation of lysine 27 on histone H3 (H3K27me2/3) (Fig. 1) catalyzed by PRC2 (*P*olycomb *R*epressive *C*omplex-*2*; the SET domain protein KMT6^SET-7^ is the subunit that specifically methylates histones) (Freitag 2017, Wiles and Selker 2017). This facultative heterochromatin mark is densely enriched over predominantly gene-rich regions of the genome (Fig. 1) to repress gene expression (Jamieson et al. 2013, Basenko et al. 2015, Dumesic et al. 2015). In *N. crassa*, H3K27me2/3 is enriched over subtelomeric chromosomal regions that are positionally dependent on the presence of telomere repeats, although chromosome-internal, position independent peaks of H3K27me2/3 are readily observed (Jamieson et al. 2013, 2016, 2018, Basenko et al. 2015, Klocko et al. 2016). In other fungi, large stretches of H3K27me3 enrichment can be observed along core chromosome arms (Connolly et al. 2013, Schotanus et al. 2015, Cook et al. 2020, Carlier et al. 2021, Soyer et al. 2021, Zhang et al. 2021, Kramer et al. 2022). For example, the fusion of multiple subtelomeric domains, which form the four chromosomes in *F. graminearum*, result in large chromosome-internal domains of H3K27me3 (Connolly et al. 2013), while H3K27me3 represses genes in the dynamic AGRs regions in *V. dahliae* (Cook et al. 2020, Kramer et al. 2022) and across accessory chromosomes in *Z. tritici* or *F. oxysporum* (Schotanus et al. 2015, Fokkens et al. 2018). Interestingly, the deposition of the H3K27me2/3 mark varies with the protein subunits associated with PRC2, as loss of PRC2-associated proteins NPF (*Neurospora* p55 ortholog) or PAS (*P*RC2 *A*ccessory *S*ubunit) abolishes subtelomeric H3K27me2/3 in *N. crassa*, while the conserved protein EPR-1 is known to “read” the H3K27me2/3 mark for gene repression; in support, deletion of the EPR-1 homolog in *F. graminearum*, BP1, phenocopies loss of KMT6 and BP1 directly binds DNA to stabilize nucleosomes for enhancing transcriptional repression (Jamieson et al. 2013, Klocko et al. 2016, McNaught et al. 2020, Wiles et al. 2020, Tang et al. 2021). Genomic regions that display properties of both constitutive and facultative heterochromatin are typically not observed (Galazka et al. 2016, Klocko et al. 2016), yet some species-specific exceptions occur. For instance, the genome of *Z. tritici* contains large domains enriched for both H3K27me2/3 and H3K9me3, while only a few larger interspersed heterochromatic regions in *V. dahliae* or only the telomeres of *N. crassa* are enriched for both marks (Schotanus et al. 2015, Klocko et al. 2016, Cook et al. 2020). In addition, a newly emerging yet understudied facultative heterochromatic mark required for repressing gene expression in *N. crassa* and *Fusarium fujikuroi* is di- or tri-methylation of lysine 36 on histone H3 (H3K36me2/3), which is catalyzed by the SET-domain containing histone methyltransferase ASH1 (Bicocca et al. 2018, Janevska et al. 2018). In *N. crassa*, ASH1-specific H3K36me2 is enriched over lowly expressed genes that are also enriched with H3K27me2/3, and loss of ASH1 causes altered enrichment of H3K27me2/3 (Bicocca et al. 2018). Similarly, in *F. fujikuroi*, ASH1 methylates H3K36 to establish facultative heterochromatin at subtelomeres; ASH1 deletion increases subtelomeric H3K27me3 enrichment, yet genes at the subtelomeres become unstable (Janevska et al. 2018). These data argue that ASH1-specific H3K36me2/3 is epistatic to the action of the PRC2 complex and highlighting the need for additional study on the dynamics of facultative heterochromatin marks in multiple fungal species.

**Figure 2.**
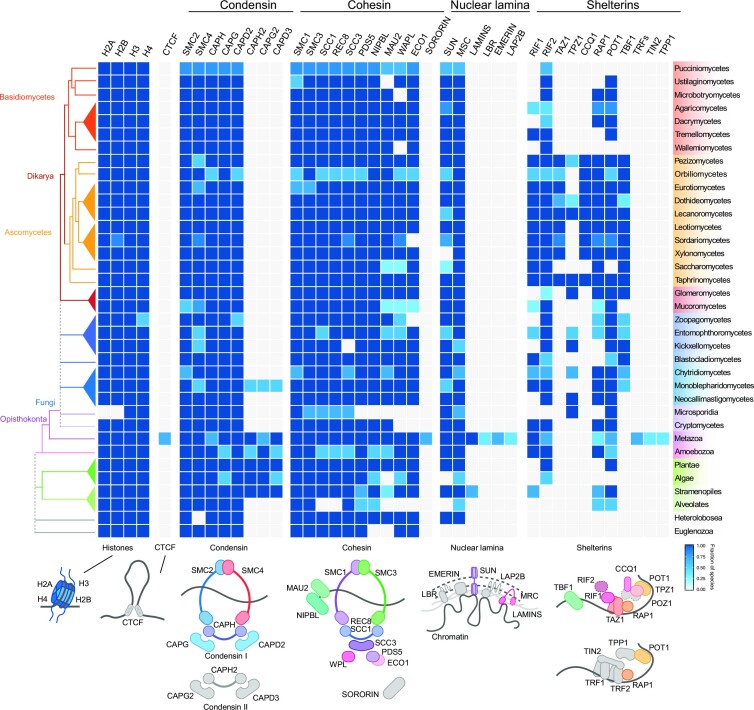
The protein landscape of chromatin organization in fungi. Phylogenetic profile of 41 well-studied proteins known to impact chromatin organization encoded in 88 eukaryotes. These proteins are divided into the core histone proteins, CTCF, condensin subunits (type I and II), cohesin subunits, nuclear envelope-associated proteins, and shelterin-associated proteins (see Table S2, Supporting Information, for specific gene information). Gene orthologs encoding most of the proteins involved in genome organization are conserved in fungi, with the exceptions of CTCF proteins associated with loop formation, condensin II for chromosome territories, Lamins at the nuclear periphery, and multiple components of the shelterin complex for telomere protection/organization. The heatmap color code reflects the fraction of species of a specific taxonomic lineage in which orthologs were found, and gray boxes indicate the absences of that specific protein. Known phylogenetic relationships between the different fungal lineages and other taxonomic groups (right) are shown on the left. Schematic representations that summarize the composition of the analyzed protein complexes are shown on the bottom; colored subunits depict presence in fungi, while gray colored subunits depict absence. Methods for ortholog searches are detailed in Supplemental File 1.

Another subclass of AT-rich isochores with distinct functions in fungal genomes are found at the telomeres, which, similar to other eukaryotes, delineate and protect chromosome ends (Rahnama et al. 2021). Like those in higher metazoans, fungal telomeres require telomerase and a shelterin complex (Smogorzewska and de Lange 2004, Nandakumar and Cech 2013, Jamieson et al. 2018, Yadav et al. 2021). Telomerase uses a ribonucleic acid template to synthesize short DNA repeats ([5′ TTAGGG]_n_) onto the chromosome ends (Szostak and Blackburn 1982, Greider and Blackburn 1985, 1989, Jain and Cooper 2010, Qi et al. 2013), while the shelterin complex protects the single stranded telomeric repeats from being degraded and/or recognized as a double strand DNA (dsDNA) break (Jain and Cooper 2010, Zinder et al. 2022). In the mammalian shelterin complex, the proteins TRF1 (*T*elomeric *R*epeat-binding *F*actor *1*), TRF2 (*T*elomeric *R*epeat-binding *F*actor *2*), and POT1 (*P*rotection *O*f *T*elomeres protein *1*) bind the telomeric repeats while TIN2 (*T*ERF1-*I*nteracting *N*uclear factor *2*), TPP1 (*T*INT1, *P*TOP, *P*IP*1* complex), and RAP1 (*R*epressor *A*ctivator *P*rotein *1*) mediate protein intracomplex interactions (de Lange 2005, Palm and de Lange 2008, Myler et al. 2021). However, only RAP1 and POT1 have clear fungal homologs (Fig. 2). In fact, fungal shelterin complexes are quite diverse, as highlighted by the divergence of shelterin complex members in two closely related yeast species (Fig. 2) (Steinberg-Neifach and Lue 2015, Erlendson et al. 2017, Xue et al. 2017). In *S. cerevisiae*, RAP1 directly recognizes telomeric dsDNA and recruits RIF1/2 (*R*AP1 *I*nteracting *F*actor *1/2*) to regulate telomere length, while TBF1 (*T*TAGGG *B*inding *F*actor *1*), a yeast ortholog of human TRF1, binds to single stranded telomeric DNA to antagonize improper telomere lengthening (Bilaud et al. 1996, Li et al. 2000, Hediger et al. 2006, Pitt et al. 2008, Kabir et al. 2010, Kaizer et al. 2015, Irie et al. 2019). In contrast, the *S. pombe* shelterin complex contains the proteins TAZ1 (*T*elomere-*A*ssociated in *Schi*Z*osaccharomyces pombe 1*; a homolog of mammalian TRF1/2), RAP1, RIF1, POT1, TPZ1 (T*elomeres* P*rotection protein in Schi*Z*osaccharomyces pombe 1*), and CCQ1 (*C*oiled-*C*oil Quantitatively enriched protein *1*) for protecting telomeric DNA and silencing subtelomeric chromatin (Cooper et al. 1997, Baumann and Cech 2001, Kanoh and Ishikawa 2001, Miyoshi et al. 2008, Fujita et al. 2012, Deng et al. 2015, Zofall et al. 2016, Irie et al. 2019). In filamentous fungi, the shelterin complexes of *N. crassa* and other ascomycetes appear to be an amalgam of budding and fission yeast shelterins, based on protein conservation (Fig. 2). *Neurospora* encodes a TAZ1 homolog to bind to dsDNA and a TBF1 homolog to bind to single stranded [5′ TTAGGG]_n_ telomeric DNA; TAZ1 (labeled TRF1 in Galazka et al. 2016, Klocko et al. 2016) is a functional homolog of mammalian TRFs, i.e. restricted to ascomycetes (Fig. 2). Other fungal species may employ unique shelterin complex proteins, including the basidiomycete corn smut pathogen *Ustilago maydis* that uses the telomere-repeat binding proteins UmTay1, UmPot1, and UmTrf2 (Yu et al. 2018, 2020, Zahid et al. 2022). Variability at yeast telomere repeats might partially explain this shelterin complex diversity in fungal species, given that different proteins would be required to recognize alternative telomere repeat sequences or differing telomere lengths (Malyavko et al. 2014, 2019, Sepsiova et al. 2016, Červenák et al. 2017, 2021, Lue 2021) For example, *S. cerevisiae* telomeres have the sequence [T(G)_2–3_(TG)_1–6_], while *S. pombe* telomeres have [TTACAG_2–5_] (Červenák et al. 2021). In contrast, *N. crassa* and other filamentous fungi use the canonical [5′ TTAGGG_n_] repeat found in metazoans to recruit the shelterin complex (Casas-Vila et al. 2015, Erlendson et al. 2017), arguing filamentous fungi could be more appropriate models for studies on telomere protection in higher organisms.

## Conserved chromosomal features in euchromatic regions in fungal genomes

Euchromatic genes have the potential to be transcriptionally active, but presumably control of gene expression can be driven by transcription factor recruitment, deposition of specific histone post-translational modifications, or even the 3D genome organization. Many fungi share similar epigenetic marks as higher eukaryotes over actively transcribed genes, including the extensive acetylation of histone proteins and the di- or tri-methylation of lysine 4 on histone H3 (H3K4me2/3) (Pokholok et al. 2005, Lewis et al. 2009, Anderson et al. 2010, Smith et al. 2010, Xiong et al. 2010, Bicocca et al. 2018, Zhu et al. 2019, Courtney et al. 2020). As in metazoans, H3K4me3 is primarily enriched at promoter regions (Fig. 1), while H3K4me2 is predominantly deposited across gene bodies (Liu et al. 2005, Pokholok et al. 2005, Cemel et al. 2017, Zhu et al. 2019). H3K4me2/3 is catalyzed by the conserved COMPASS complex, which contains the SET-domain protein KMT2^SET-1^ as its catalytic subunit, but the composition of associated subunits can vary (Miller et al. 2001, Roguev et al. 2001, Freitag 2017). All four histone proteins within euchromatic nucleosomes are also substantially acetylated (Smith et al. 2010), with multiple lysine residues being subject for acetylation, including lysine 27 on histone H3 (H3K27ac; Fig.   1) (Bicocca et al. 2018). H3K27ac is also observed in *M. oryzae*, where loss of H3K27me3 and gain in H3K27ac increases transcription of genes necessary for plant infection (Zhang et al. 2021). In general, acetylation appears dense over gene bodies and intergenic spacing, but little acetylation is found in AT-rich isochores, as deacetylase complexes, such as the HCHC in *N. crassa*, act upon heterochromatic regions to remove acetyl groups to induce chromatin condensation (Honda et al. 2012, 2016).

The distribution of these “activating” histone modifications can influence the formation of nucleosomes in euchromatin by altering the association of histone proteins with DNA, thereby changing the accessibility of the underlying DNA (Bannister and Kouzarides 2011, Smolle and Workman 2013). Euchromatin accessibility has been assessed by ATAC-seq (Assay for Transposase Accessible Chromatin-sequencing). For example, *Neurospora* displays highly accessible chromatin regions (ACRs) that are often found upstream of genes: most are small, but a subset of these intergenic ACRs are large (>2000 bp), presumably for multiple transcription factors to control of expression of ACR-proximal genes; these open chromatin regions are enriched with acetylation of H3K27 to possibly “open” the chromatin (Ferraro et al. 2021). Euchromatin is characterized by a more open nucleosome conformation that would also allow the underlying DNA to be more accessible. Specifically, the promoter DNA of expressed genes is typically devoid of histone proteins, forming a nucleosome free region that allows transcription factor binding to *cis*-regulatory elements for the recruitment of the RNA Polymerase II machinery for transcription initiation (Bai and Morozov 2010, Radman-Livaja and Rando 2010, Struhl and Segal 2013). Nucleosome-free regions (NFR) at promoters with more accessible chromatin have been widely observed in fungi, including *S. cerevisiae* (Oberbeckmann et al. 2019), *N. crassa* (Klocko et al. 2019, Ferraro et al. 2021), *C. albicans* (Jenull et al. 2020), *Aspergillus niger* (Huang et al. 2021), and *Ustilaginoidea virens* (Chen et al. 2021). Histone variants that alter nucleosome composition are also known to impact chromatin accessibility across eukaryotic genomes (Talbert and Henikoff 2010, 2017, Henikoff and Smith 2015). For example, the placement of the histone variant H2A.Z, which replaces H2A in the histone octamer, is enriched at the first nucleosome immediately downstream of the transcription start site of actively transcribed genes, possibly functioning to impact transcription by altering the chromatin structure at fungal promoters (Dong et al. 2018, Chen and Ponts 2020, Martire and Banaszynski 2020). Additionally, modifying the position of nucleosomes in the open reading frames of genes or within intergenic regions can impact the accessibility or composition of chromatin. Nucleosome positions are altered by chromatin remodelers, and in fungi, well-studied chromatin remodelers include the SWI-SNF complex and the AAA-ATP member DIM-1/CATP, the latter of which impacts the enrichment of H3K9me3 and 5^m^C in intergenic regions in euchromatin (Cha et al. 2013, Klocko et al. 2019, Kamei et al. 2021, Wiles et al. 2022).

## The spatial organization of fungal chromatin

During interphase, the nucleosomes package DNA into 10 nanometer (nm) chromatin fibers, a beads on a string conformation that in turn may aggregate further into 30 nm chromatin fibers (Bernardi 2015, Maeshima et al. 2016, Hansen et al. 2018b, Wako et al. 2020). Presumably, these chromatin fibers would be spatially organized in the nucleus to facilitate proper genome function, including the initiation of transcription to enable timely gene expression (Cook 1999, Misteli 2007). To facilitate this nuclear organization, the chromatin fibers in eukaryotic genomes form a series of subnuclear structures of hierarchically increasing sizes—in fungi, these range from chromatin loops to the Rabl chromosome conformation (Box 1)—that both allow critical genome features to aggregate into nuclear euchromatic/heterochromatic compartments and prevent the formation of knots (or other nonviable DNA strand folding). This hierarchical organization of fungal chromatin folding allows euchromatic genes to have the potential to dynamically associate in spatially close proximity, thereby forming nonrandom interactions or higher-order 3D structures critical for gene expression. However, one can speculate that the large-scale folding of chromatin fibers is also necessary to compact the fungal genome in the nucleus to ensure the proper functioning of the DNA templated processes necessary for viability in an identical manner to the role of genome organization in metazoan genome function (Misteli 2007, Cavalli and Misteli 2013, Sexton and Cavalli 2015). It should be noted, however, that most genome organization experiments in fungi are derived from Ascomycetes, and consequently additional work in Basidiomycetes as well as in earlier diverging fungi will be needed to determine which aspects of the hierarchical genome organization detailed below are conserved across fungi.

### Chromatin loops/globules

Chromatin loops (often called “globules”) are among the most prevalent 3D structures in metazoan genomes (Kadauke and Blobel 2009, Lieberman-Aiden et al. 2009, Heger et al. 2012). Here, chromatin loops occur when two loci physically separated on the linear chromosome associate, and the intervening chromatin condenses. In metazoans, these ∼300 kilobases (kb) chromatin loops may functionally influence gene expression by facilitating interactions between distant regulatory sequences, such as enhancers and silencers, although the possibility exists that chromatin loops only form to structurally organize metazoan genomes (Cavalli and Misteli 2013, Rao et al. 2014, 2017, Dekker and Heard 2015). Two critical components in the formation of chromatin loops in humans are cohesin and condensin I, which are architectural DNA-binding protein complexes important for DNA replication and chromosome folding, as well as for condensing chromosomes for meiosis and mitosis (Green et al. 2012, Hirano 2012, Haarhuis et al. 2017, Rao et al. 2017, Davidson and Peters 2021, Hoencamp et al. 2021, Jeppsson et al. 2022). In the cohesin complex, the *S*tructural *M*aintenance of *C*hromosomes (SMC)-*1*, SMC-3, Scc1, and Scc3 proteins form the core machinery for chromatin looping (Dorsett 2007, Peric-Hupkes and van Steensel 2008, Skibbens 2019). In addition, five to six accessory proteins are responsible for dynamically loading or unloading the core cohesin complex onto chromatin and activating the cohesin ATPase activity (Haarhuis et al. 2017, Davidson and Peters 2021, Yoshida et al. 2022). The loading and activation of the SMC complex onto chromatin in budding yeast is facilitated by the yeast homologs Scc2 and Scc4, as signaled by the acetyltransferase Eco1, while cohesin dissociation from chromatin is mediated by WAPL and PDS5 (Ciosk et al. 2000, Rolef Ben-Shahar et al. 2008, Chan et al. 2012, Murayama and Uhlmann 2014, Çamdere et al. 2015, Haarhuis et al. 2017, Petela et al. 2018). Together, SMC proteins comprise a ring-shape structure in the cohesin complex through which chromatin is actively extruded, thereby forming chromatin loops (Fudenberg et al. 2016, Ganji et al. 2018, Bauer et al. 2021). Chromatin extrusion continues until a boundary element is encountered, which both signals for cessation of extrusion mechanism and the anchoring of the resulting chromatin loop (Ganji et al. 2018). Cohesin remains associated with chromatin through interphase, potentially to stabilize loops, until mitosis dependent Scc1 cleavage occurs (Uhlmann et al. 1999, Nasmyth 2001, Murayama and Uhlmann 2014). Borders of chromatin loops are typically demarcated in metazoan Hi-C datasets by the visualization of a “focus” or point of enriched contacts off diagonal (Rao et al. 2014). These strong interactions at loop bases are often centered over binding sites for the CTCF (CCCTC binding factor) (Fudenberg et al. 2016, Brackley et al. 2018, Banigan and Mirny 2020, Davidson and Peters 2021). CTCF-mediated loops are known to insulate nearby active and repressive chromatin regions by blocking further chromatin extrusion (de Wit et al. 2015, Sanborn et al. 2015). Here, two CTCF proteins bind to convergently oriented asymmetric 14 bp sequences and dimerize to form a loop of chromatin in the genomes of higher eukaryotes (Ong and Corces 2014, Rao et al. 2014, de Wit et al. 2015, Nora et al. 2017). Consequently, depletion of CTCF disrupts loop formation in many cases (Zuin et al. 2014, Nora et al. 2017, Xu et al. 2021).

**Figure 3.**
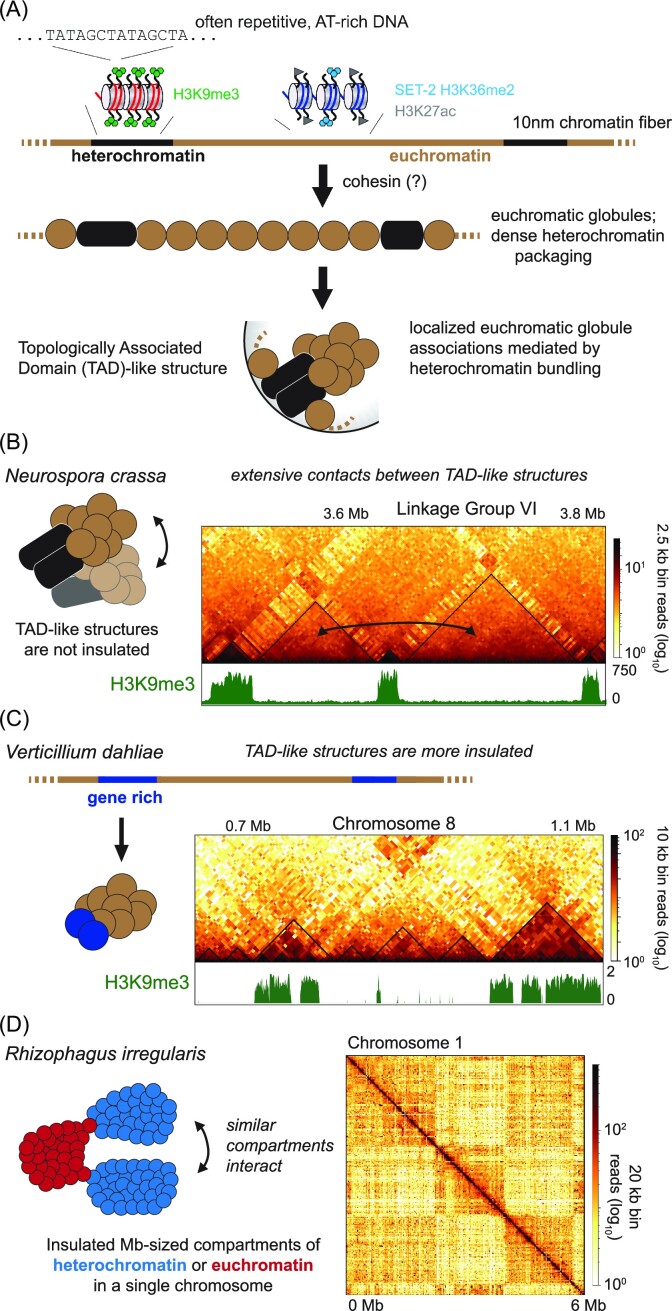
The formation of regional structures in several fungal organisms. In filamentous fungi, TAD-like structures generally form through hierarchical clustering of local chromatin structures. (A) A generalized schematic of the folding of chromatin from the “beads-on-a-string” model of the 10-nm fiber in which DNA is bound to histone proteins to form nucleosome, to the formation of loops/globules with the action of cohesin, to the folding of those globules into TAD-like/regional globule clusters in the fungal genome. The folding of TAD-like structures inferred from Hi-C contact data in (B) *N. crassa* (Rodriguez et al. 2022) and (C) *V. dahliae* (Torres et al. 2023). (D) The compartmentalization of heterochromatic (stronger interactions) and euchromatic regions can be observed in the genome of *Rhizophagus irregularis* (Yildirir et al. 2022). Methods for Hi-C data processing and image generation are detailed in Supplemental File 1.

The cohesin core complex and most accessory proteins are highly conserved throughout eukaryotes (Fig. 2), so it is reasonable to expect that chromatin loop formation occurs by a loop extrusion mechanism in fungi as well (Fig. 3A). In fungi, the role of cohesin has been only examined in the fission yeast *S. pombe*, where in a temperature-sensitive cohesin mutant strain the ∼40 kb-sized globules are no longer visible across the genome (Mizuguchi et al. 2014, Tanizawa et al. 2017). Chromatin loops/globules have been also observed in filamentous fungi. In *N. crassa* slightly larger (∼60–80 kb) globules are readily visible in Hi-C datasets (Rodriguez et al. 2022). Given the conservation of cohesin subunits in these filamentous fungi (Fig. 2), cohesin should also act across these genomes. However, it is possible that cohesin and its activity are not fully conserved throughout all fungal lineages. In Microsporidia, almost all cohesin complex components are absent (Fig. 2), suggesting that these spore-forming unicellular fungal parasites evolved distinct mechanisms to topologically organize their genome. Further, WAPL and Eco1 seem to be absent in fungi belonging to the Zoopagomycetes (Fig. 2), suggesting novel mechanisms for signaling loop dissociation might exist in these obligate soil nematode parasites. Interestingly, and in contrast to cohesin subunits, the gene encoding CTCF is restricted to higher metazoan species, which typically have larger genomes. One can speculate that the CTCF bound to the base of a chromatin loop anchors, and possibly stabilizes, the larger-sized globules/loops in metazoans (Li et al. 2020, Pugacheva et al. 2020). Other boundary elements apart from CTCF have been found in plants (Dong et al. 2017, Xie et al. 2019), *Drosophila melanogaster* (Ramírez et al. 2018), *Caenorhabditis elegans* (Anderson et al. 2019), and humans (Anania et al. 2022, Valton et al. 2022). However, the specific boundary elements restricting chromatin loop size by repressing cohesin loop extrusion in fungi have not been fully characterized (Mizuguchi et al. 2014, Schalbetter et al. 2019, Rodriguez et al. 2022). Fungi may employ diverse mechanisms to negatively regulate loop formation, including the enrichment of convergent genes that delineate loop “boundaries” in *S. pombe* (Mizuguchi et al. 2014), or the incorporation of AT-rich, repetitive heterochromatic isochores in the genome, as observed in *N. crassa* (Rodriguez et al. 2022) and *E. festucae* (Winter et al. 2018). While future experiments are needed to assess the requirement of cohesin for forming chromatin loops in filamentous fungi (Schalbetter et al. 2019), the hypothesis that constitutive heterochromatic regions possibly act as loop anchors would render CTCF or similar boundaries unnecessary in some fungi.

### Topologically associated domains

Topologically associated domains (TADs) are 3D chromosomal structures that regionally organize the genome by subdividing chromatin compartments (Dixon et al. 2012, Acemel and Lupiáñez 2023) suggesting that a single TAD may contain several chromatin loops or become visible when multiple loops are averaged over a cell population (Dekker and Heard 2015, Hansen et al. 2018a) (Fig. 3). TADs in metazoans are typically megabase-sized genomic regions in which chromatin is more apt to contact yet is insulated from chromatin outside of the TAD (Lieberman-Aiden et al. 2009, Dixon et al. 2012). In metazoan Hi-C datasets, TADs appear as triangles of increased contact probability immediately off-diagonal, with few distant contacts with chromatin beyond the TAD border (Fig. 3). Interestingly, TAD borders in metazoans are often enriched for housekeeping genes, tRNA genes, and retrotransposons (Dixon et al. 2012). However, it is currently unclear if TADs are primarily structural, i.e. TAD formation organizes the genome in the nucleus, or are essential for genome function (Beagan and Phillips-Cremins 2020), possibly required to regulate gene expression by increasing the contact probability of distant enhancers/silencers with cognate promoters (Flavahan et al. 2016, Dixon et al. 2018). While altered TAD borders may cause misregulation of gene expression in some human cancers (Flavahan et al. 2016, Taberlay et al. 2016, Valton and Dekker 2016, Dixon et al. 2018, Akdemir et al. 2020), multiple datasets report only minimal changes in gene expression when individual TADs are altered (Rao et al. 2017, Ghavi-Helm et al. 2019). Additionally, TADs change little through interphase of the cell-cycle, but TADs are consistently lost upon entry into mitosis when chromosomes are locally condensed, and are reformed before entry to G1, suggesting TADs are primarily structural in nature (Naumova et al. 2013, Abramo et al. 2019).

TAD formation in higher metazoans appears to require the action of condensin complexes. Specifically, the SMC complexes condensin type I and II are known to shape individual chromosomes by forming higher order looping structures (Hagstrom and Meyer 2003, Hirano 2012). In both complexes, the yeast homologs SMC2 and SMC4 form a highly conserved core ring structure (Schleiffer et al. 2003), but individual condensin types can be distinguished by other subunits: condensin I contains CAPH, CAPG, and CAPD2, while condensin II has CAPH2, CAPG2, and CAPD3 (Ono et al. 2003, Schleiffer et al. 2003). Condensin I has the greatest impact during the mitotic phase of the cell cycle, where it laterally compacts sister chromatids by forming smaller supercoiled chromatin loops (Kimura and Hirano 1997, Hagstrom and Meyer 2003, Green et al. 2012, Golfier et al. 2020, Kong et al. 2020). Condensin II affects high order chromosome organization during all cell cycle stages, as it is found in the nucleus during S phase; during mitosis, condensin II may involve the formation of large chromatin loops, in conjunction with condensin I activity, for establishing a “nested loop architecture” critical for sister chromatid/homologous chromosome pairing (Yu and Koshland 2005, Ono et al. 2013, Kong et al. 2020).

Emerging evidence from multiple species suggests that filamentous fungi have TAD-like structures analogous to metazoan TADs (Mizuguchi et al. 2014, Eser et al. 2017, Tsochatzidou et al. 2017, Winter et al. 2018, Schalbetter et al. 2019, Rodriguez et al. 2022). Fungal TAD-like structures are several hundred kilobases in size and the euchromatin internal to these TAD-like structures is more apt to contact (Dixon et al. 2012, Galazka et al. 2016, Rodriguez et al. 2022). In *N. crassa*, TAD-like structures were originally termed regional globule clusters (RGCs) named for the aggregation of several ∼40 kb euchromatic globules into larger, compact structures ∼250 kb in size that could be interpreted as a large chromatin aggregates analogous to metazoan TADs (Fig. 3B). RGCs display extensive yet random internal euchromatic contacts that are not restricted from outside chromatin, as strong inter-RGC contacts readily occur, arguing proteins insulating internal euchromatin are not encoded in *N. crassa* (Rodriguez et al. 2022). RGCs are flanked by constitutive heterochromatic regions to delineate RGC borders. The clustering of heterochromatin regions, possibly through liquid–liquid phase separation (LLPS) condensates (Larson et al. 2017), may act as RGC anchor (Fig. 3B) in an analogous manner to CTCF at chromatin loops (Rodriguez et al. 2022); consequently, cohesin would act specifically to form the smaller globules internal to and comprising this TAD-like structure. Similar patterns of TAD-like structures have been observed in *S. cerevisiae*, where globule structures are delimited by transcriptionally active genes that are often in a convergent orientation (Tsochatzidou et al. 2017, Schalbetter et al. 2019). Additionally, TAD-like structures can be seen in *E. festucae*, where RIP’d AT-rich heterochromatic regions strongly interact to form large structures to compact chromatin (Winter et al. 2018), and in the fungal pathogen *Puccinia striiformis*, which may form uninsulated RGCs across each chromosome arm (Xia et al. 2022). Another example of TAD-like structures is found in *V. dahliae* and related *Verticillium* species where Hi-C datasets display TAD-like structures with increased internal chromatin contact probabilities and few intrachromosomal contacts beyond the TAD-like structure boundaries (Fig. 3C) (Torres et al. 2023). In contrast to the situation in *N. crassa*, (differentially) expressed genes occur at, or in proximity to, TAD-like boundaries in *V. dahliae* and *E. festucae*, suggesting that TAD-like structures in these fungi are necessary for proper gene expression. Additional evidence from yeasts suggests that TAD-like structures are critical for other genome functions apart from gene expression, such as repressing recombination or promoting genome evolution (Mizuguchi et al. 2014, Tsochatzidou et al. 2017, Gu et al. 2022). For example, TADs may be essential for fungal chromosome replication during S-phase of the cell cycle, as ∼200-kb TAD-like structures across the *S. cerevisiae* genome separate clusters of early or late timed origins of replication across its 16 chromosomes (Eser et al. 2017).

### Organization of interspersed constitutive heterochromatic regions

**Figure 4.**
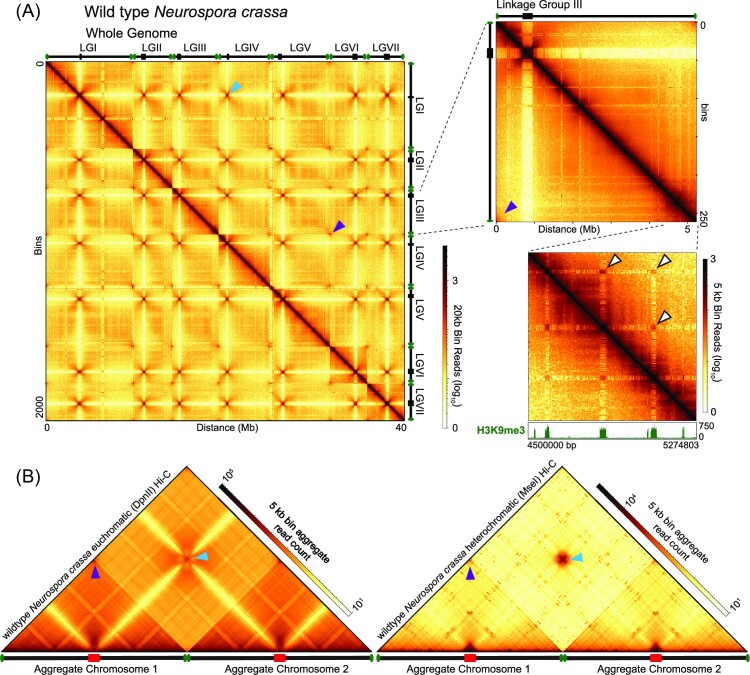
Genome organization of the filamentous fungus *N. crassa*. The genome organization of the seven chromosomes comprising the entire *N. crassa* genome is characterized by interchromosomal centromeric contacts, as well as interchromosomal contacts between telomeres in a Rabl chromosome conformation. Individual chromosomes have strong telomeric interactions, while centromeric chromatin strongly self-interacts yet is isolated from other genomic foci. Interestingly, the strongest long-range interactions occur between H3K9me3-enriched constitutive heterochromatic regions. (A) Corrected *in situ* Hi-C data of combined DpnII (euchromatin-specific) and MseI (heterochromatin-specific) Hi-C data (Rodriguez et al. 2022) at 20 kb resolution, showing the interactions across the entire genome (all seven chromosomes indicated on the top and right of the Hi-C contact map), one chromosome (Linkage Group III), and a zoomed image of the right arm of LG III. Blue arrowheads indicate interchromosomal centromere interactions, purple arrowheads indicate inter- or intrachromosomal telomeric interactions, and white arrowheads indicate strong intrachromosomal heterochromatic interactions. ChIP-seq track of H3K9me3 enrichment shows regions of constitutive heterochromatin. (B) Aggregate chromosome plots, at 5 kb resolution, of DpnII (euchromatin-specific, left) or MseI (heterochromatin-specific, right) *in situ* Hi-C data (Rodriguez et al. 2022). Two aggregate chromosome schematics are below the Hi-C contact map, with centromeres denoted by red boxes and telomeres shown by green ovals. Blue arrowheads indicate interchromosomal centromere interactions and purple arrowheads indicate intra- or interchromosomal telomeric interactions. Methods for Hi-C data processing and image generation are detailed in Supplemental File 1.

AT-rich isochores comprising constitutive heterochromatin can be found embedded throughout fungal chromosomes as well as at centromeres and telomeres (Lewis et al. 2009, Winter et al. 2018, Seidl et al. 2020). *Neurospora crassa* has approximately 300 AT- and H3K9me3-enriched isochores interspersed throughout the genome, which range in size from <1 to ∼400 kb (Galazka et al. 2016, Klocko et al. 2016). These regions readily associate in the nucleus, as exceptionally strong contacts between silent chromatin regions both within a chromosome and across chromosomes are frequently observed in fungal Hi-C datasets (Fig. 4), arguing that the clustering of constitutive heterochromatic regions in the fungal nucleus may be particularly important for the proper chromosome conformation in fungi. However, it is readily apparent in fungal Hi-C datasets that any interspersed constitutive heterochromatic region has the potential to interact, as uniformly strong contacts between all H3K9me3-marked silent regions are readily observed within Hi-C data derived from a population of fungal nuclei (Galazka et al. 2016, Winter et al. 2018, Rodriguez et al. 2022). Moreover, the chromatin inside every constitutive heterochromatic region strongly and consistently interacts across the entire length of that silent region, with the strongest interactions occurring on the level of individual nucleosomes, suggesting that silent chromatin forms dense globule-like structures consisting of a stochastic nucleosome aggregation (Galazka et al. 2016, Winter et al. 2018, Rodriguez et al. 2022). At the highest resolutions, dense globules are visible at the boundaries between heterochromatic and euchromatic regions, implying the formation of 3D chromatin structures to prevent heterochromatin spread (Rodriguez et al. 2022). However, the loss of the known constitutive heterochromatin machinery has little impact on the folding of individual heterochromatic regions. In *Neurospora*, deletion of the gene encoding the KMT1^DIM-5^ histone methyltransferase or its cognate binding partner HP1 reduces the dense internal compaction of heterochromatic regions and leads to reduced contacts between the euchromatin bordering these silent regions (Galazka et al. 2016). This suggests that folding of constitutive heterochromatic regions is dependent on proper deposition of different chromatin modifications and that the primary role of H3K9me3 and HP1 is to compact individual silent regions, thereby restricting contacts even between distant heterochromatic regions (Galazka et al. 2016, Zenk et al. 2021). Notably, AT-rich DNA forms few contacts with the surrounding euchromatin despite active and silent chromatin being in close proximity on the linear chromosome (Galazka et al. 2016, Klocko et al. 2016, Rodriguez et al. 2022), suggesting the heterochromatin-internal nucleosomes are isolated from active chromatin in fungal nuclei. Similarly, H3K9me3-enriched AT-rich sequences in *V. dahliae* and AT-rich isochores in *E. festucae* appear to be insulated from euchromatic contacts (Winter et al. 2018, Seidl et al. 2020)

Despite the overall segregation of heterochromatic and euchromatic DNA, recent work has shown that contacts that form between active and silent chromatin may regulate fungal gene expression (Rodriguez et al. 2022). Specifically, in *N. crassa*, small “bands” of strong interactions between H3K9me3-marked constitutive heterochromatic regions and select genes in more GC-rich genomic regions, which are possibly marked with a unique combination of histone post-translational modifications, are readily observed at the highest resolution Hi-C datasets (Fig. 1) (Rodriguez et al. 2022). Many of these genes display drastic changes in gene expression when constitutive heterochromatin is compromised (e.g. in a Δ*dim-5*/Δ*kmt1* mutant strain) (Rodriguez et al. 2022). The possibility of constitutive heterochromatin regulating gene expression has been observed previously (Yang et al. 2014) and may not be limited to *Neurospora* as several hundred genes that significantly change expression have been observed in *V. dahliae* upon loss of the lysine 9-specific histone methyltransferase *Dim-5* (Kramer et al. 2021).

To segregate heterochromatic genomic loci from those that are actively transcribed, and possibly to prevent aberrant RNA synthesis of silent chromatin, heterochromatin clusters at the nuclear membrane in virtually all eukaryotic nuclei (Gonzalez-Sandoval and Gasser 2016, Solovei et al. 2016, Falk et al. 2019). In mammals, most heterochromatin associates with lamin filaments and additional anchor proteins to form LADs (*L*amina-*A*ssociated *D*omains) at the nuclear envelope (Guelen et al. 2008, Briand and Collas 2020). Mammalian proteins required for LAD formation include the lamin B receptor, Emerin, and *L*amina-*A*ssociated-*P*olypeptide *2*-b (LAP-2), all of which contain a LEM (*L*AP-2, *E*merin, *M*AN1) domain capable of binding chromatin at the nuclear envelope (Lin et al. 2000, Wagner and Krohne 2007, Buchwalter et al. 2019). However, fungi do not encode these proteins (Fig. 2), nor has the LEM domain been observed in any fungal proteins (Wagner and Krohne 2007, Koreny and Field 2016). However, fungi encode two general classes of integral nuclear membrane proteins that facilitate heterochromatin interactions with the nuclear membrane: MSC and SUN proteins (Koreny and Field 2016). Members of the MSC family of proteins include the integral membrane proteins Src1/Heh1, Heh2, Man1, and Lem2 (Brachner et al. 2005, King et al. 2006, Wagner and Krohne 2007, Grund et al. 2008, Mekhail and Moazed 2010, Taddei and Gasser 2012). All MSC proteins contain N-terminal LEM-like and MSC (*M*an1-*S*rc1p *C*-terminal) domains in their primary structures. In *S. pombe*, Lem2 facilitates the anchoring of heterochromatic regions to the nuclear envelope, where the chromatin silencing machinery, including histone deacetylase complexes targeted to the telomeres, are recruited for chromatin repression (Sugiyama et al. 2007, Barrales et al. 2016, Hirano et al. 2020). Further, individual heterochromatic regions require specific proteins for nuclear envelope association: centromeres require Csi1 and telomeres use Dsh1 and Bqt3 (or Bqt4) (Barrales et al. 2016, Harr et al. 2016, Ebrahimi et al. 2018). The SUN (Sad-1, Unc-84) protein family similarly mediates heterochromatin interactions with the nuclear membrane (Tzur et al. 2006). Specifically, the *S. cerevisiae* SUN protein Mps3 associates with Sir silencing proteins, including the Sir4–Sir3 complex that binds deacetylated histone H4 in silent chromatin (Bupp et al. 2007, Mekhail and Moazed 2010, Taddei and Gasser 2012, Harr et al. 2016). These observations highlight a direct nuclear membrane-heterochromatin contact being important for genome organization. The MSC and SUN proteins are also widely conserved across filamentous fungi (Fig. 2), implying similar mechanisms might also be employed in these species to tether heterochromatic regions to the nuclear membrane.

### Chromatin compartmentalization in fungi

Euchromatin and heterochromatin in metazoans typically segregates into two distinct nuclear compartments: the euchromatic (active) “A” and heterochromatic (silent) “B” compartments. This compartmentalization of chromatin is readily observed as a “checkerboard” pattern in Hi-C contact maps (Fig. 3D). The functional interpretation of this pattern is that genomic regions that have a similar transcriptional activity (e.g. heterochromatic regions that are silent) are spatially interacting within the nucleus (Lieberman-Aiden et al. 2009, Dixon et al. 2012, Rao et al. 2014, Dong et al. 2017, Rowley et al. 2017, Nichols and Corces 2021). Further studies have demonstrated that chromatin in each compartment physically associates: in the A-compartment euchromatin interacts in the central nucleus region, while in the B-compartment heterochromatin associates at the nuclear periphery (Lieberman-Aiden et al. 2009, Rao et al. 2014, 2017, Buchwalter et al. 2019, Beagan and Phillips-Cremins 2020). Mechanistically, this segregation may occur due to the aggregation of heterochromatic regions, possibly through LLPS, at the nuclear membrane causing euchromatin to associate in the nucleus center (Larson et al. 2017, Falk et al. 2019), or due to the forces emerging from the activity of DNA-templated proteins in euchromatin forcing the segregation of silent chromatin to the nuclear periphery (Mahajan et al. 2022). In contrast to the extensive compartmentalization seen in metazoan Hi-C datasets, few fungi have evidence of prominent compartmentalization. To date, only the arbuscular mycorrhizal fungus *Rhizophagus irregularis*, a member of the Glomeromycetes clade, displays clear A/B compartments (Fig. 3D) (Xia et al. 2022, Yildirir et al. 2022). In contrast, other fungi display minimal chromatin compartmentalization, possibly reflecting the presence of smaller heterochromatic regions integrated among larger euchromatin domains (Xia et al. 2022). However, segregation of fungal chromatin in a manner analogous to A/B compartments, where heterochromatic regions aggregate yet are separated from euchromatic TAD-like structures, has been observed in the Hi-C datasets of multiple fungal species, including *S. cerevisiae* (Duan et al. 2010), *S. pombe* (Mizuguchi et al. 2014), *N. crassa* (Galazka et al. 2016, Rodriguez et al. 2022), *E. festucae* (Winter et al. 2018), *A. bisporus* (Hoencamp et al. 2021), and *V. dahliae* (Seidl et al. 2020). Presumably, interactions between heterochromatic regions, even when the small AT-rich isochores across fungal genomes are formed into heterochromatin, could be crucial for phase separation into the active “A” and silent “B” compartments (Falk et al. 2019). Chromatin compartmentalization is also supported by historical electron microscopy data, which shows clusters of densely stained heterochromatin, which are often at the nuclear periphery but can be in the nucleus center, interspersed with lightly stained euchromatin (Shatkin and Tatum 1959), thus arguing the chromatin composition organizes the nuclear genome in fungi.

### Organization of fungal chromosomes into a Rabl conformation

**Figure 5.**
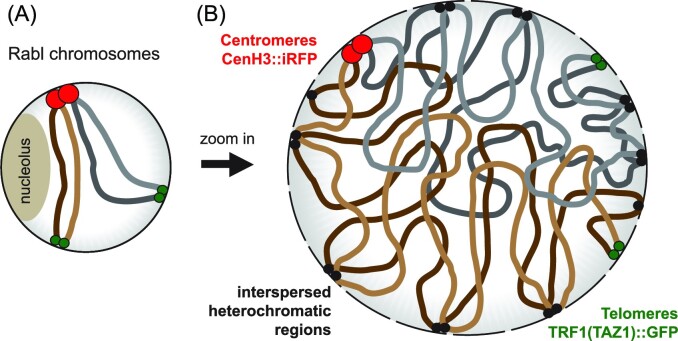
The formation of the Rabl chromosome conformation in fungal nuclei. (A) A schematic representation of two chromosomes (colored brown and gray for distinction) in the Rabl chromosome conformation (Mizuguchi et al. 2015), where centromeres (red circles) cluster on one side of the nucleus and the telomeres (green circles) cluster on the opposite side; the nucleolus is a distinct structure apart from these interphase chromosomes. (B) Detailed schematic of how interspersed heterochromatic regions (black circles) facilitate chromatin associating with the nuclear membrane and the compartmentalization of active and silent chromatin. Each chromosome forms a weak territory, with more intrachromosomal contacts, but some interchromosomal interactions can occur.

The compartmentalization of the heterochromatic centromeres and telomeres of fungal chromosomes onto the nuclear membrane would facilitate the formation of Rabl chromosome conformations within the fungal nucleus (Box 1) (Duan et al. 2010, Kim et al. 2017, Schalbetter et al. 2019, Hoencamp et al. 2021). Rabl chromosome conformation is typically characterized by the clustering of centromeres on one side of the nuclear envelope and chromosomal arms extending outwards towards the opposing nuclear periphery on which the (sub)telomeres associate (Fig. 5A; e.g. Jin et al. 2000). Microscopic (e.g. Guacci et al. 1994, Laroche et al. 1998, Jin et al. 2000, Goto et al. 2001, Gasser 2002, Schober et al. 2008) as well as Hi-C experiments (Duan et al. 2010, Kim et al. 2017, Schalbetter et al. 2019, Hoencamp et al. 2021) also confirmed that *S. cerevisiae* organizes its 16 chromosomes in Rabl conformation during interphase, with a centromere cluster in proximity to the spindle pole body while the 32 (sub)telomeres associate nonrandomly in four to eight foci at the nuclear membrane opposite the centromere bundle (Bystricky et al. 2004, Duan et al. 2010, Therizols et al. 2010, Kim et al. 2017, Schalbetter et al. 2019). Similarly, *S. pombe* organizes its three chromosomes into a Rabl structure (Mizuguchi et al. 2014). In filamentous fungi, the Rabl conformation was initially observed in *N. crassa* Hi-C experiments (Galazka et al. 2016, Klocko et al. 2016, Rodriguez et al. 2022). These data were corroborated by fluorescent microscopy of *N. crassa* nuclei in which a single centromere focus and three to four telomeric foci associate with the nuclear membrane (Fig. 5, Box 1). Additional Hi-C data from a plethora of filamentous fungi show that the centromeres contact independent of—and distinct from—telomere clustering and thus confirmed the existence of Rabl chromosomes, including in ascomycetes [*Penicillium oxalicum* (Li et al. 2022), *E. festucae* (Winter et al. 2018), *V. dahliae* (Seidl et al. 2020, Torres et al. 2023), *Candida albicans* (Burrack et al. 2016), *Fusarium verticillioides* (Yao et al. 2023), and *Cladosporium fulvum* (Zaccaron et al. 2022)] and basidiomycetes [*Puccinia polysora* (Liang et al. 2022), *Puccinia graminis* (Sperschneider et al. 2021, Henningsen et al. 2022), *Austropuccinia psidii* (Edwards et al. 2022), and *Agaricus bisporus* (Hoencamp et al. 2021)] (Table S1, Supporting Information). Interestingly, *R. irregularis* does not show clear centromere bundling for organizing its chromosomes, but it does seem to exhibit telomere bundling (Yildirir et al. 2022). Thus, these observations collectively suggest that the vast majority of fungi exhibit at least some of the features associated with Rabl-like chromosomal conformation.

One of the most prominent features observed in Hi-C contact maps of species employing a Rabl chromosome conformation is the strong interchromosomal interactions indicative of centromere bundling (Fig. 4). Centromere bundles manifest as dark “spots” between chromosomes in whole genome contact maps, as observed in yeasts (*S. cerevisiae* and *S. pombe*), *N. crassa* (Fig. 4), *V. dahliae*, and *E. festucae*, among others (Mizuguchi et al. 2014, Belton et al. 2015, Kim et al. 2017, Tanizawa et al. 2017, Winter et al. 2018, Seidl et al. 2020, Rodriguez et al. 2022). The size of the interchromosomal centromere contacts is dependent on whether the centromeres in that species are point (small intercentromere interactions) or regional (large intercentromere interactions) centromeres (Belton et al. 2015, Galazka et al. 2016, Tanizawa et al. 2017, Yadav et al. 2018a, b, Seidl et al. 2020, Rodriguez et al. 2022). Work in budding yeast has shown that centromeres bundle at the spindle pole body during interphase, an interaction which may require the centromeres to associate with microtubules as the application of nocodazole, a toxin that disrupts microtubule assembly, leads to reduced centromere clustering (Jin et al. 2000, Goto et al. 2001). The yeast CBF3 complex, which deposits the centromere specific histone variant Cse4 in centromeric DNA, also contains the critical kinetochore protein Ndc10p, highlighting the connection between centromere clustering and microtubule binding (Lechner and Carbon 1991, Yan et al. 2018). Surprisingly, few interactions between the heterochromatic centromeres and other interspersed heterochromatic regions are observed in *N. crassa*, even when heterochromatic features are in close proximity on the linear chromosome (Galazka et al. 2016, Rodriguez et al. 2022). This argues that the centromeric bundle is refractory to interacting with other heterochromatic regions, implying that the centromere bundle forms a dense, compact structure that isolates centromeric DNA (Figs 4 and 5). Specificity for centromere bundling may be derived from deposition of CenH3 into centromeric nucleosomes, as CenH3 enrichment is only observed at the centromeres in *N. crassa* and other fungi (Smith et al. 2012, Galazka et al. 2016, Seidl et al. 2020). Presumably, kinetochore proteins that specifically associate with these centromeric histone variants might play a role in establishing and maintaining the centromere bundles at the nuclear membrane across filamentous fungi, in a manner similar to that of yeast (Pidoux and Allshire 2004, Westermann et al. 2007, Biggins 2013). Centromere clustering may also require some general property of heterochromatin independent of H3K9me3 deposition, as yeasts lacking H3K9me3 and SET-domain histone methyltransferases still have extensive interchromosomal centromere bundling, suggesting centromere clusters could rely on the silent chromatin in these regions forming LLPS condensates.

Increased contacts between telomeres that indicate clustering of chromosome ends are another prevalent feature of most fungal Hi-C interaction maps (Fig. 4), and strong interactions originating at chromosome ends in Hi-C datasets can extend ∼200 kb internal to the chromosome into the subtelomeres (Fig. 4) (Mizuguchi et al. 2014, Galazka et al. 2016, Klocko et al. 2016, Kim et al. 2017, Tanizawa et al. 2017, Rodriguez et al. 2022). The relative position and distance between telomere bundles in the nucleus are governed by the chromosomal arm length, the position of the centromere, and the nuclear volume (Bystricky et al. 2004, Therizols et al. 2010). Individual telomeres may also have a unique landscape of chromatin modifications. For instance, in *Neurospora* these chromosome ends are the only loci in the genome enriched for both H3K9me3 and H3K27me2/3 (Klocko et al. 2016, Jamieson et al. 2018). However, the terminal H3K9me3-enriched telomere repeats minimally interact with nearby interspersed facultative or constitutive heterochromatic regions (Rodriguez et al. 2022). Importantly, no experimental data thus far has observed the colocalization of both centromeres and telomeres in the same nuclear region in wild type cells (Fig.   4), suggesting that mechanisms exist that independently organize these chromosomal structures. One possibility is that fungi may co-opt facultative heterochromatin to restrict (sub)telomeric contacts, thereby ensuring proper genome organization. In support, the Δ*set-7* (Δ*kmt6*) *N. crassa* strain devoid of H3K27me2/3 can have microscopic colocalization of centromeres and telomeres when facultative heterochromatin is compromised (Klocko et al. 2016). In Hi-C datasets, this loss of H3K27me2/3 reduces subtelomeric interactions and causes general genome organization disorder consistent with reduced interactions between the subtelomeres and the nuclear membrane (Klocko et al. 2016). However, only subtelomeric H3K27me2/3 is critical for nuclear membrane interactions, as a Δ*npf* (*Neurospora* p55 homolog) strain that loses subtelomeric H3K27me2/3 exhibits genome disorder consistent with compromised subtelomere interactions despite internal H3K27me2/3 enrichment being maintained (Klocko et al. 2016). Thus, current data seems to suggest that the unique chromatin landscape at the centromeres and telomeres ensure the isolation of these chromosomal features in a Rabl conformation despite both exhibiting properties of constitutive heterochromatin.

Loss of shelterin complex members eliminates telomere clustering and impacts the localization of telomeres to the nuclear periphery, as increased numbers of telomere foci are observed in loss of function shelterin mutants (Palladino et al. 1993, Chikashige and Hiraoka 2001, Kanoh and Ishikawa 2001). The heterodimeric Yku complex (Yku70/80) may be required for telomere clustering, as Yku70/80 anchors telomeres to the nuclear envelope (Laroche et al. 1998, Hediger et al. 2002), but also plays a role in shielding telomeric ends from shortening or fusion, and in telomere silencing (Boulton and Jackson 1998, Polotnianka et al. 1998, Ponnusamy et al. 2008). In filamentous fungi, recent work elucidated the role of an unexpected protein contributing to telomere tethering. In the Neurospora *dim-3* strain, which encodes a mutant allele of Importin ɑ/Karyopherin ɑ, some telomere foci no longer associate with the nuclear membrane, and a *dim-3* strain exhibits a drastically altered genome organization in a manner consistent with compromised telomere anchoring (Galazka et al. 2016). This phenotype may be indirectly caused by an increase in nuclear volume, which under normal situations would physically constrain telomere anchoring: *dim-3* nuclei consistently have a larger nuclear membrane diameter than the nuclei in wild-type strains (Galazka et al. 2016). Consistently, previous work in metazoans suggest that Importin ɑ/Karyopherin ɑ is necessary to constrict nuclear volumes (Levy and Heald 2010). Further experiments aimed at elucidating the proteins necessary for telomere–nuclear membrane anchoring, based on the groundwork established in yeast systems, should prove fruitful to uncover if filamentous fungi use similar mechanisms for telomere anchoring.

Quantitative computational models suggest that the specific tethering of heterochromatic chromosomal regions, including centromeres and telomeres to distinct nuclear landmarks, is sufficient to explain higher order organization of fungal genomes (Tjong et al. 2012, Wong et al. 2012). For example, in budding yeast, the complex Rabl nuclear organization emerges in computational models in which chromosomes are allowed to form random contacts, yet chromosomes are constrained by the tethering of chromosomal features to the nuclear envelope, by the distances between telomeres, and by the colocalization of functionally related loci (Tjong et al. 2012, Wong et al. 2012). Therefore, these geometrical constraints alone are sufficient to explain highly organized nuclear genome organization, including the Rabl-like chromosomal conformation, in *S. cerevisiae* (Tjong et al. 2012). Despite the genomes of filamentous fungi being larger and often containing more chromosomes, the bundling of centromeres, telomeres, and interspersed heterochromatic regions at the nuclear periphery would be expected to drive the Rabl chromosome organization in a similar manner.

One corollary effect of a Rabl conformation in fungal nuclei is that chromosomes cannot form distinct territories, a property of higher metazoan genomes in which each chromosome occupies a defined space in the nucleus (Manuelidis 1985, Cremer and Cremer 2001, 2010, Tanabe et al. 2002, Parada et al. 2004). In humans, chromosomal territories are evident by an enhanced intrachromosome contact frequency, and minimal interchromosomal interactions (Lieberman-Aiden et al. 2009, Imakaev et al. 2012, Falk et al. 2019, Hoencamp et al. 2021). Eukaryotes encoding a complete condensin II complex form these territories, arguing that the presence of condensin II either strengthens chromosomal territories or suppresses Rabl conformation formation (Hoencamp et al. 2021). Specifically, deletion of the condensin II subunit CAPH2 in human cells promotes the formation of a Rabl-like chromosome conformation by increasing interchromosomal and trans-centromeric contacts while lowering lengthwise compaction of chromosomes (Hoencamp et al. 2021, Yoshida et al. 2022). However, nuclear architecture is more variable when longer evolutionary time scales are considered (Hoencamp et al. 2021). Specifically, fungal genomes do not encode condensin II accessory subunits (Fig. 2), as previously noted in *S. cerevisiae, S. pombe*, and *N. crassa* (Hudson et al. 2009, Hirano 2012, Hoencamp et al. 2021, Rodriguez et al. 2022), and consequently strong chromosome territories rarely form in fungal nuclei. Thus, the formation of a Rabl chromosome conformation (Fig. 5) in fungi is near ubiquitous. In this model, each chromosome exhibits extensive interchromosomal contacts (Fig. 4) that could be necessary for proper genome function, including the regulation of fungal gene expression. However, several intriguing observations directly contrast the Rabl chromosome model being applicable to all fungi. First, in *R. irregularis*, no clear Rabl chromosomes can be observed in Hi-C experiments (Fig. 3D) (Yildirir et al. 2022) yet condensin II orthologs are absent (Fig. 2), suggesting novel proteins facilitate chromatin compartmentalization into novel subnuclear structures. Further, orthologs of the condensin II accessory subunits CAPH2, CAPG2, and CAPD3 are present in species of the Monoblepharidomycetes clade (Fig. 2), suggesting that chromosome territories may exist in these taxa, but no Hi-C data currently exists to test this hypothesis.

## Concluding remarks and future research

The spatial organization of the eukaryotic nuclear genome is closely linked to its biological functions (Lieberman-Aiden et al. 2009, Sexton and Cavalli 2015, Bonev and Cavalli 2016, Hoencamp et al. 2021). Here, we addressed the occurrence, formation, and functional implications of the spatial organization on the nuclear genome in fungi. Yeasts have been important model systems to study nuclear genome organization in the last decades, but only recently data on the composition and organization of chromatin in more diverse fungi became available. Based on these, we sought to summarize and discuss structures homologous to those found in model eukaryotes and examined the protein complexes that are implicit in establishing these structures. We argued that the folding of chromatin fibers in fungi is similarly hierarchical as in other eukaryotes, ranging from small-scale chromatin loops of a few kilobases to large-scale subdomains comprising hundreds of kilobases that segregate chromatin into A or B compartments. The self-interacting domains similar to metazoan TADs have been observed in yeasts (Duan et al. 2010, Mizuguchi et al. 2014, Tsochatzidou et al. 2017, Schalbetter et al. 2019), and TAD-like structures that regionally organize the genome are prevalent in most studied filamentous fungi. However, the precise nature of the boundary or insulator regions that allow the loading of cohesin or restrict chromatin extrusion remains to be examined in detail. Furthermore, based on experimental data from several diverse fungi, we argued that Rabl chromosome conformation is the hallmark of fungal genome organization, and the independent bundling of centromeres and telomeres drives the overall nuclear organization of the fungal genome. Implicit in this hierarchical organization is that different “levels” are interconnected and that changes in local chromatin organization have significant impact on the global nuclear organization, e.g. in formation of heterochromatic structure at centromeres and telomeres and *vice versa*. In addition to the critical link between gene expression and chromatin folding, several conserved DNA-templated processes may be directly tied to genome organization, including DNA replication and repair that can alter genome and nuclear organization. DNA repair events can contribute to fungal evolution, as the occurrence of a double stranded DNA break and its concomitant repair onto DNA strands spatially close in 3D may be the first step in structurally varying a species’ genome (Zhang et al. 2012, Hanson et al. 2021, Huang and Cook 2022). Thus, DNA-templated processes in the nucleus are often influenced by the organization of the genome, yet improper functioning of these genomic functions can feedback and alter genome organization, highlighting the intricate interconnection between chromosome conformations and genome function. While we here strived to paint a complete picture on the genome organization in fungi, it is important to note that detailed, high-resolution datasets on chromosomal conformation as well as chromatin composition and organization are only available for very few model fungal species. Even though the hierarchical organization of nuclear genome organization is largely conserved between the fungal species examined to date, we also highlighted intriguing differences between species, including the species-specific characteristics defining TAD-like structures. Recent work comparing the position of TAD-like structures and conservation of TAD boundaries between fungal strains and species of the same genus suggests that there is limited TAD variation (Torres et al. 2023), which is reminiscent of observations in other eukaryotes (Rao et al. 2014, Harmston et al. 2017, Rowley et al. 2017, Krefting et al. 2018, Fudenberg and Pollard 2019, Liao et al. 2021, McArthur and Capra 2021). These data favor a hypothesis where TAD-like structures are generally conserved in related fungi but may be less conserved when greater fungal diversity is considered. Nevertheless, in *Drosophila* species, rearrangements occur predominantly at TAD boundaries and not in TAD bodies (Liao et al. 2021, Wright and Schaeffer 2022), suggesting that TAD-like structures play important roles for the evolution of genome organization. Therefore, comparative studies that systematically probe genome organization throughout the fungal lineage will help deepen understanding of the establishment, conservation, and functional implications of genome organization. We, therefore, advocate that exploring genome organization in the context of the extensive fungal biodiversity will be essential to uncover in the future how nuclear organization impacts fungal genome function and evolution.

Box 1: **The history of deciphering genome organization**

Historically, studies on genome organization date to the late 1800s when Carl Rabl made his seminal observations on chromosome organization in eukaryotic nuclei (Cremer and Cremer 2006, 2010). Using light microscopy, he observed that centromeres are located on one side of the nucleus and the telomeres are found at the opposing side, an observation that was consistently maintained during the cell cycle. Further advances in the 1950s, made by examining cells with electron microscopy, elucidated the partitioning of the active and silent chromatin: the densely compacted heterochromatin predominantly localizes at the nuclear periphery while the more-open euchromatin is mostly found in nucleus center (Shatkin and Tatum 1959, Cremer and Cremer 2006). The advent of fluorescent microscopy allowed individual genomic features to be examined (Renz 2013), either with dyes that generally stained the DNA (e.g. DAPI), fluorescent probes to examine individual loci [e.g. fluorescent in situ hybridization (FISH) experiments], fluorescently tagged proteins to examine the nuclear location and dynamics of proteins *in vivo* in live cells, or fluorescently labeled antibodies that highlight the localization of individual proteins in the nucleus in fixed cells. These advances elucidated the alternating banding pattern of euchromatin and heterochromatin in *Drosophila* salivary gland polytene chromosomes (Bridges 1938, Zhimulev et al. 2014), how some genes colocalize with RNA Polymerase II in possible cotranscriptional hubs (Schoenfelder et al. 2010), and the Rabl chromosome conformation that organizes fungal genomes into weak chromosome territories; in a Rabl conformation, the centromeres from each chromosome cluster into a single focus at the nuclear periphery, while at a different inner nuclear membrane location, the telomeres of each chromosome associate into several foci (Funabiki et al. 1993, Guacci et al. 1994, Goto et al. 2001, Gasser 2002, Klocko et al. 2016) (Box 1 figure). However, these methods are limited by the number of fluorescent dyes with unique emission wavelengths and the resolution of microscopic images (Lichtman and Conchello 2005, Carlton 2008), thereby restricting the number of loci and/or proteins that could be examined at the same time and the resolution by which we can study the interactions of individual proteins and/or colocalization of genes.

Recently, these challenges have been addressed by the introduction of chromosome conformation capture (3C)-based experiments, which revolutionized the field of nuclear genome organization (Dekker et al. 2002). In 3C, chromatin is cross-linked with nonspecific cross-linking agents (e.g. formaldehyde), underlying DNA is digested with restriction enzymes, and interacting genomic loci are ligated into a single DNA molecule. Traditional 3C uses a PCR reaction with target-specific primers to show interacting genomic loci. Coupling 3C to high-throughput sequencing (Hi-C) allows researchers to examine on a genome-wide scale how genomic regions interact (Lieberman-Aiden et al. 2009). Typically, Hi-C results are displayed as a two dimensional (2D) heatmap, with the x- and y-axes plotting the genomic location on the chromosome(s), and the intensity of color showing the strength of interactions as measured by the contact probability (Fig. 4), although the 3D chromosome folding can also be modeled from Hi-C data (Galazka et al. 2016, Oluwadare et al. 2019), thereby allowing researchers to infer the folding of chromosomes in exquisite detail and derive functional and structural mechanisms for how this organization occurs.

**Box 1 Figure.**
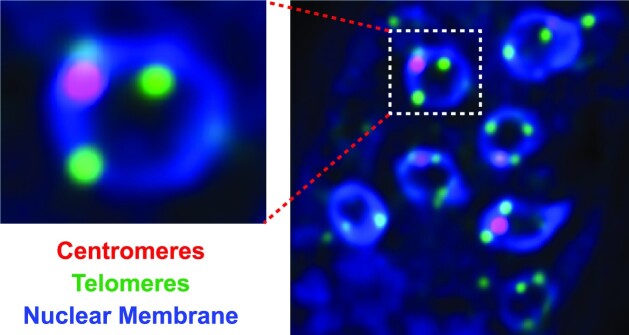
Fluorescent microscopy can assess specific chromosomal features in fungi. The clustering of centromeres, which is independent of the colocalization of telomeres, and the association of these features with the nuclear membrane is instrumental to the formation of a Rabl chromosome conformation in fungal nuclei. Fluorescent micrograph of conidia from a *N. crassa* strain expressing CenH3::iRFP (red) to illuminate centromeres, TZA1::GFP (previously reported as TRF1::GFP; green) to highlight telomeres, and ISH1::BFP (blue) demarcate the nuclear membrane (Klocko et al. 2016). The image on the left is an enhanced image of a single nucleus from the conidia image on the right. Methods for imaging are detailed in Supplemental File 1.

## Supplementary Material

fuad021_Supplemental_Files
