# Supplementary material for: Nuclear genome organization in fungi: from gene folding to Rabl chromosomes
Source: FEMS Microbiol Rev. 2023 May 17;47(3):fuad021. doi: 10.1093/femsre/fuad021 (PMC10246852; doi:10.1093/femsre/fuad021)
Supplement: fuad021_Supplemental_Files [file fuad021_supplemental_files.zip › SupplementaryFile1_clean.docx]

*Supplementary Material for:*

**Nuclear Genome Organization in Fungi: From Gene folding to Rabl Chromosomes**

David E. Torres^1,2^, Andrew T. Reckard^3^, Andrew D. Klocko^3^*^#^, Michael F. Seidl^1^*^#^

**Supplemental Methods**

*Imaging analysis*

Imaging of the *Neurospora crassa* strain N6224 (*ish-1::foTagBFP2::hph::LoxP; hpo-TagRFP-T::hph::loxP; CenH3::iRFP670::hph::LoxP; Trf-1::GFP::hph::LoxP;* Δ*sad-2::nat1*) was performed under standard imaging conditions (conidia from N6224 were resuspended in a 50% glycerol solution, and 3 µL was spotted on glass microscope slides and covered with a glass coverslip) (Klocko *et al.* 2016) on a Leica Thunder Imaging System; composite and deconvoluted images of GFP, BFP, and iRFP were cropped in Adobe Photoshop.

*Hi-C data analysis*

Paired-end sequence fastq files of Hi-C data were downloaded from the NCBI GEO (*N. crassa*) or the Sequence Read Archive (SRA; *R. irregularis*) using the program fastq-dump, mapped with bowtie2 (Langmead and Salzberg 2012) or BWA mem (*V. dahliae,* (Li and Durbin 2009)), and processed with hicExplorer (Ramírez *et al.* 2018), using the subprograms hicBuildMatrix, hicCorrect using Knight Ruiz correction for *N. crassa* and *R. irregulatis* (Knight and Ruiz 2013) or Iterative Correction (ICE) correction for *V. dahliae* (Imakaev *et al.* 2012), hicPlotMatrix, and hicPlotTADs. Aggregate read count matrices for *N. crassa* Hi-C data were built with Juicer (Rao *et al.* 2014; Hoencamp *et al.* 2021) using fastq files of DpnII (euchromatin-specific) or MseI (heterochromatin-specific) Hi-C datasets (Rodriguez *et al.* 2022), and the resultant .mcool matrix files were displayed with hicExplorer. The contact matrix for *V. dahliae* Hi-C data was built with hicBuildMatrix (Ramírez *et al.* 2018) from DpnII (euchromatin-specific) proximity ligation Hi-C data and displayed using hicPlotMatrix.

*Chromatin Immunoprecipitation data analysis*

ChIP-seq data was mapped to version 14 of the *N. crassa* genome using bowtie2 (Langmead and Salzberg 2012), converted to a bam file and sorted with samtools (Li *et al.* 2009), and the sequence enrichment was calculated by the bamCoverage program in DeepTools (Ramírez *et al.* 2016) and displayed as a bigwig file on Integrative Genomics Viewer (IGV) (Robinson *et al.* 2011). Similar methods were used to display H3K9me3 ChIP-seq data for *V. dahliae* using the reference strain JR2 (Faino *et al.* 2015). Percent guanine-cytosine bases (%GC) was calculated for the nc12 fixed genome and displayed as a .wig file (Klocko *et al.* 2019); given the differences between versions 12 and 14 of the *N. crassa* genome only affect chromosomes (Linkage Groups) I and V (Rodriguez *et al.* 2022), the same wig file can be used for Linkage Group III images. Transposon location bed files were displayed with IGV, with the locations modified from (Nguyen *et al.* 2022).

*Phylogenetic profile of chromatin topology associated proteins in fungi*

Database

To identify proteins involved in chromatin topology in fungi, we used 88 eukaryotic (predicted) proteomes (Supplementary Table 2). Proteomes of non-fungal taxa (15) were obtained from an in-house dataset described previously (Deutekom *et al.* 2019). The vast majority of fungal proteomes were obtained from the Joint Genome Institute (JGI) Mycocosm fungal reference database (https://mycocosm.jgi.doe.gov/mycocosm/home). Additionally, the predicted proteomes of *Austropuccinia psidii* (Edwards *et al.* 2022) and *Hemileia vastratix* (Tobias *et al.* 2022) were manually added after prediction of protein-coding genes by Augustus with default settings (Stanke *et al.* 2006).

HMM model assignment

Hmmersearch (HMMER v.3.1 (Eddy 2011)) was used with in-house profile hidden Markov models (HMMs), Pfam 31.0 (Finn *et al.* 2016), and histone profiles (https://www.ncbi.nlm.nih.gov/research/HistoneDB2.0/) to search the in-house database for homologous sequences. Briefly, in house profile HMMs were generated by obtaining diverse eukaryotic sequences for lamins (Kollmar 2015), shelterin subunits (Finn *et al.* 2016; Myler *et al.* 2021), SUN and SMC (Koreny and Field 2016). The condensin (I, II), cohesin, and CTCF profile HMMs were kindly provided by Dr. Jolien J. E. van Hooff, Université Paris-Saclay, CNRS, AgroParisTech, Ecologie Systématique Evolution, Orsay, France. Homologous sequences in our fungal database for each protein family were aligned using MAFFT v7.453 --localpair --maxiterate 1000 (Katoh and Standley 2013), trimmed to remove non-informative sites using ClipKit v.1.3.0 –smartgap (Steenwyk *et al.* 2020). The final profile HMMs were generated using hmmbuild with default settings (Eddy 2011). To identify the final set of homologs, we used hmmersearch with -E 1e-10, --domE 1e-10, --incE 1-e-10, --incdomE 1-e-10 as settings. Matching proteins for each profile HMM in our database were extracted, aligned using MAFFT v7.453 --auto --maxiterate 1000 (Katoh and Standley 2013) and multiple sequence alignments were trimmed using trimal v.1.4.1 --gt 0.1 (Capella-Gutiérrez, Silla-Martínez and Gabaldón). We built a phylogenetic tree for each alignment using IQ-TREE v.2.2.0.3 (Nguyen *et al.* 2015) with LG model (--mset LG (Le, Dang and Gascuel 2012)) and 1,000 ultrafast bootstraps (--B 1000, (Minh *et al.* 2020)). The presence of similar Pfam domains for each protein were verified in a multiple sequence alignment using hmmerscan and the Pfam database with default settings (Eddy 2011; Finn *et al.* 2016). Finally, we only considered sequences as an ortholog if after the profile HMMs search the tree topology and the Pfam domain supports a monophyletic relationship with well-characterized eukaryotic proteins. Protein sequences for the orthologous families are available at Zenodo doi:10.5281/zenodo.7635847.

**Data Availability**

Hi-C data is publicly available at either the Gene Expression Omnibus (GEO; for *N. crassa* [accession number GSE173593]) (Rodriguez *et al.* 2022) or the Sequence Read Archive (SRA; for *R. irregularis* [accession number PRJNA748024] and *V. dahliae* [accession PRJNA641329]) (Seidl *et al.* 2020; Yildirir *et al.* 2022) at the National Center of Biotechnology Information (NCBI) at the National Institutes of Health, USA. Publicly available Chromatin Immunoprecipitation-sequencing (ChIP-seq) from *N. crassa* wild type (WT) datasets of H3K9me3 (merged from GSE68897 and GSE98911), H3K27me2/3 (merged from GSE82222 and GSE100770), H3K27ac (GSE118495), and H3K4me3 (GSE121356) are available at the GEO from published manuscripts (Galazka *et al.* 2016; Jamieson *et al.* 2016; Klocko *et al.* 2016, 2019, 2020; Bicocca *et al.* 2018; Zhu *et al.* 2019). Publicly available ChIP-seq from *V. dahliae* wild type (WT) datasets of H3K9me3 (Cook *et al.* 2020) is accessible from the NCBI/SRA under BioProject PRJNA592220.

**Reference**

Bicocca VT, Ormsby T, Adhvaryu KK *et al.* ASH1-catalyzed H3K36 methylation drives gene repression and marks H3K27me2/3-competent chromatin. *Elife* 2018;**7**:e41497.

Capella-Gutiérrez S, Silla-Martínez JM, Gabaldón T. trimAl: trimming in large-scale phylogenetics analyses. *trimal.cgenomics.org*.

Cook DE, Martin Kramer H, Torres DE *et al.* A unique chromatin profile defines adaptive genomic regions in a fungal plant pathogen. *Elife* 2020; **9**:e62208.

Deutekom ES, Vosseberg J, van Dam TJP *et al.* Measuring the impact of gene prediction on gene loss estimates in Eukaryotes by quantifying falsely inferred absences. *PLoS Comput Biol* 2019;**15**:e1007301.

Eddy SR. Accelerated Profile HMM Searches. *PLoS Comput Biol* 2011;**7**:e1002195.

Edwards RJ, Dong C, Park RF *et al.* A phased chromosome-level genome and full mitochondrial sequence for the dikaryotic myrtle rust pathogen, *Austropuccinia psidii*. *bioRxiv* 2022:2022.04.22.489119.

Faino L, Seidl MF, Datema E *et al.* Single-Molecule Real-Time sequencing combined with optical mapping yields completely finished fungal genome. *MBio* 2015;**6** (4):e00936-15

Finn RD, Coggill P, Eberhardt RY *et al.* The Pfam protein families database: towards a more sustainable future. *Nucleic Acids Res* 2016;**44**:D279–85.

Galazka JM, Klocko AD, Uesaka M *et al.* Neurospora chromosomes are organized by blocks of importin alpha-dependent heterochromatin that are largely independent of H3K9me3. *Genome Res* 2016;**26**:1069–80.

Imakaev M, Fudenberg G, McCord RP *et al.* Iterative correction of Hi-C data reveals hallmarks of chromosome organization. *Nat Methods* 2012;**9**:999–1003.

Jamieson K, Wiles ET, McNaught KJ *et al.* Loss of HP1 causes depletion of H3K27me3 from facultative heterochromatin and gain of H3K27me2 at constitutive heterochromatin. *Genome Res* 2016;**26**:97–107.

Katoh K, Standley DM. MAFFT multiple sequence alignment software version 7: improvements in performance and usability. *Mol Biol Evol* 2013;**30**:772–80.

Klocko AD, Uesaka M, Ormsby T *et al.* Nucleosome positioning by an evolutionarily conserved chromatin remodeler prevents aberrant DNA methylation in *Neurospora*. *Genetics* 2019;**211**:563–78.

Klocko AD, Ormsby T, Galazka JM *et al.* Normal chromosome conformation depends on subtelomeric facultative heterochromatin in *Neurospora crassa*. *Proc Natl Acad Sci U S A* 2016;**113**:15048–53.

Klocko AD, Summers CA, Glover ML *et al.* Selection and characterization of mutants defective in DNA Methylation in *Neurospora crassa*. *Genetics* 2020;**216**:671–88.

Knight PA, Ruiz D. A fast algorithm for matrix balancing. *IMA J Numer Anal* 2013;**33**:1029–47.

Koreny L, Field MC. Ancient eukaryotic origin and evolutionary plasticity of nuclear lamina. *Genome Biol Evol* 2016;**8**:2663–71.

Kollmar M. Polyphyly of nuclear lamin genes indicates an early eukaryotic origin of the metazoan-type intermediate filament proteins. *Sci Rep* 2015;**5**:10652

Langmead B, Salzberg SL. Fast gapped-read alignment with Bowtie 2. *Nat Methods* 2012;**9**:357–9.

Le SQ, Dang CC, Gascuel O. Modeling protein evolution with several amino acid replacement matrices depending on site rates. *Mol Biol Evol* 2012;**29**:2921–36.

Li H, Durbin R. Fast and accurate short read alignment with Burrows–Wheeler transform. *Bioinformatics* 2009;**25**:1754–60.

Li H, Handsaker B, Wysoker A *et al.* The Sequence Alignment/Map format and SAMtools. *Bioinformatics* 2009;**25**:2078–9.

Minh BQ, Schmidt HA, Chernomor O *et al.* IQ-TREE 2: New models and efficient methods for phylogenetic inference in the genomic era. *Mol Biol Evol* 2020;**37**:1530–4.

Myler LR, Kinzig CG, Sasi NK *et al.* The evolution of metazoan shelterin. *Genes Dev* 2021;**35**:1625–41.

Nguyen L-T, Schmidt HA, von Haeseler A *et al.* IQ-TREE: a fast and effective stochastic algorithm for estimating maximum-likelihood phylogenies. *Mol Biol Evol* 2015;**32**:268–74.

Nguyen D, Peona V, Unneberg P *et al.* Transposon- and genome dynamics in the fungal genus *Neurospora*: Insights from nearly gapless genome assemblies. *Fungal Genet Rep* 2022;**66**:1.

Ramírez F, Bhardwaj V, Arrigoni L *et al.* High-resolution TADs reveal DNA sequences underlying genome organization in flies. *Nat Commun* 2018;**9**:189.

Ramírez F, Ryan DP, Grüning B *et al.* deepTools2: a next generation web server for deep-sequencing data analysis. *Nucleic Acids Res* 2016;**44**:W160–5.

Rao SSP, Huntley MH, Durand NC *et al.* A 3D map of the human genome at kilobase resolution reveals principles of chromatin looping. *Cell* 2014;**159**:1665–80.

Rodriguez S, Ward A, Reckard AT *et al.* The genome organization of *Neurospora crassa* at high resolution uncovers principles of fungal chromosome topology. *G3* 2022;**12**(5):jkac053.

Robinson JT, Thorvaldsdóttir H, Winckler W *et al.* Integrative genomics viewer. *Nat Biotechnol* 2011;**29**:24–6.

Seidl MF, Kramer HM, Cook DE *et al.* Repetitive elements contribute to the diversity and evolution of centromeres in the fungal genus *Verticillium*. *MBio* 2020;**11**(5):e01714-20.

Stanke M, Keller O, Gunduz I *et al.* AUGUSTUS: ab initio prediction of alternative transcripts. *Nucleic Acids Res* 2006;**34**:W435–9.

Steenwyk JL, Buida TJ 3rd, Li Y *et al.* ClipKIT: A multiple sequence alignment trimming software for accurate phylogenomic inference. *PLoS Biol* 2020;**18**:e3001007.

Tobias PA, Edwards RJ, Surana P *et al.* A chromosome-level genome resource for studying virulence mechanisms and evolution of the coffee rust pathogen *Hemileia vastatrix*. *bioRxiv* 2022:2022.07.29.502101.

Yildirir G, Sperschneider J, Malar C M *et al.* Long reads and Hi-C sequencing illuminate the two-compartment genome of the model arbuscular mycorrhizal symbiont *Rhizophagus irregularis*. *New Phytol* 2022;**233**:1097–107.

Zhu Q, Ramakrishnan M, Park J *et al.* Histone H3 lysine 4 methyltransferase is required for facultative heterochromatin at specific loci. *BMC Genomics* 2019;**20**:350.
